# Efficacy of two vaccine formulations against contagious bovine pleuropneumonia (CBPP) in Kenyan indigenous cattle

**DOI:** 10.1016/j.rvsc.2011.08.020

**Published:** 2012-10

**Authors:** Isabel Nkando, Joycelyne Ndinda, Joseph Kuria, Jan Naessens, Flora Mbithi, Christian Schnier, Michael Gicheru, Declan McKeever, Hezron Wesonga

**Affiliations:** a Kenya Agricultural Research Institute, Veterinary Research Centre, P.O. Box 32, 00902 Kikuyu, Kenya; b University of Nairobi, Faculty of Veterinary Medicine, P.O Box 29053, 00625 Nairobi, Kenya; c Kenyatta University, Faculty of Science, P.O. Box 43844, 00100 Nairobi, Kenya; d International Livestock Research Institute, Old Naivasha Road, P.O. Box 30709, 00100 Nairobi, Kenya; e Moredun Research Institute, Edinburgh, Scotland, UK

**Keywords:** Contagious bovine pleuropneumonia, *Mycoplasmamycoides* subsp. *mycoides* SC, Vaccine, Efficacy

## Abstract

A live, attenuated vaccine is currently the only viable option to control of CBPP in Africa. It has been suggested that simple modifications to current vaccines and protocols might improve efficacy in the field. In this report we compared the current vaccine formulation with a buffered preparation that maintains *Mycoplasma* viability at ambient temperature for a longer time. Groups of animals were vaccinated with the two formulations and compared with non vaccinated groups. Half of the animals in each group were challenged 3 months post vaccination, the other half after 16 months. Protection levels were measured using the pathology index, calculated from post mortem scores of lesions from animals killed during the course of clinical disease. In the challenge at 3 months post vaccination, the protection levels were 52% and 77% for the modified and current vaccine preparations, respectively. At 16 months post vaccination, the protection levels were 56% and 62% for the modified and current vaccine preparations, respectively. These findings indicate that there are no differences in protection levels between the two vaccines. Because of its longer half life after reconstitution, the modified vaccine might be preferred in field situations where the reconstituted vaccine is likely not to be administered immediately.

## 1 Introduction

Contagious bovine pleuropneumonia (CBPP) is an important disease of cattle in Africa, causing an annual economic loss estimated at US$ 2 billion (Masiga et al., 1998) due mainly to mortalities. It also affects local and international trade due to animal movement restrictions, and further losses may be incurred during eradication campaigns, as evidenced by Botswana in 1995 (Amanfu et al., 1998).

In Africa, the disease spread alarmingly during the 1990s, affecting several countries previously free from the disease (March et al., 2000) and causing greater losses in cattle than any other disease (OIE, 1995). The factors contributing to this resurgence were thought to include the collapse of veterinary services (Provost, 1996), increased and unrestricted cattle movements as a result of drought, war, or civil strife (Windsor and Wood, 1998), and poor vaccine efficacy (Rweyemamu et al., 1995; Thiaucourt et al., 2000). According to Roeder and Rweyemamu (1995), between-zone or -country contagion was basically related to cattle movements caused by trade, transhumance and social plight.

The low efficacy of the current live vaccine is a big problem for Africa, where CBPP is controlled mainly through vaccination, supplemented only by quarantine and restriction of animal movement. Policies like control through slaughter of cattle are difficult to implement in Africa for a variety of reasons, including the cost of compensation.

While vaccine coverage and protection rates of 80–90% are the target for vaccination campaigns to maintain a disease free status, current CBPP vaccines give protection rates as low as 30–60% after primary vaccination (Wesonga and Thiaucourt, 2000). This suggests that, even with high levels of cover, effective control of the disease will not be achieved with the current vaccine. Since *Mycoplasma* *mycoides* subspecies *mycoides* small colony (*Mmm*SC), the causative agent of CBPP, is pH sensitive (March et al., 2002), it has been speculated that a loss of viability occurs in the time between reconstitution and administration of the vaccine, thus contributing to the low level of protection. Modifications to the media used in growing the organism have been found to result in greater viability, and changes in the diluent used for reconstitution resulted in prolongation of viability under laboratory conditions (March et al., 2002; March, 2004). In an attempt to evaluate the usefulness of this modification in vaccine preparations, an experimental trial was conducted to compare the efficacy of the vaccine grown in buffered medium with the current vaccine preparation. Vaccinated cattle were challenged at 3 or 16 months post vaccination and compared with non-vaccinated cattle.

## 2 Materials and methods

### 2.1 Animals

Ninety male cattle (*Bos indicus* or East African Zebu) aged 2–3 years, were purchased from the Kakamega district of the Western Province, Kenya. The area is historically known to be free from CBPP and away from stock routes associated with CBPP-infected cattle. Prior to purchase, they were ear-tagged, bled and confirmed negative for CBPP antibodies using the slide agglutination test (SAT), the complement fixation test (CFT; Campbell and Turner, 1953) and the competitive enzyme linked immunosorbent assay test (cELISA; CIRAD-EMVT, France; Le Goff and Thiaucourt, 1998). They were transported to the experimental station (Veterinary Research Centre, Muguga – Kenya Agricultural Research Institute, KARI, Central Province), where they were administered with Nilzan plus cobalt® (Cooper, Nairobi, Kenya) for helminth control and vaccinated against Foot-and-Mouth disease (FMD), lumpy skin disease (LSD), blackleg and anthrax over 3 months, during which they were grazed during the day and confined in a paddock at night. The animals were handled according to KARI Animal Welfare Committee regulations.

### 2.2 Vaccine production

Standard and the modified freeze-dried batches of the T_1_44 vaccine strain of *Mmm*SC (batch numbers 02/06 and 03/06, respectively) were obtained from KARI-Veterinary Vaccine Production Centre, prepared in accordance with standard operating procedures. The *Mycoplasma* for the vaccines were grown from a validated stock of T1/44, used for commercial production. The standard vaccine was produced in Gourlay medium as described (Litamoi et al., 1996). The modified vaccine was prepared by growing the *Mycoplasma* in a HEPES-buffered system in conjunction with Gourlay medium as described (Waite and March, 2001).

Titration of *Mycoplasma* in the vaccine preparations was carried out by the standard method of microtitration and color change and calculation of the titre by using the Spearman–Karber formula (Litamoi et al., 1996). Vaccines were reconstituted in a volume of phosphate buffered saline (PBS) (for the modified vaccine) or normal saline (for the standard vaccine) provided with the vaccines, so that the final titre of *Mycoplasma* was 10^7^/ml.

### 2.3 Experimental design

The 90 cattle were divided into three randomized cohorts of 30, each of which was vaccinated with the standard vaccine, modified vaccine or diluent alone (sterile phosphate buffered saline; PBS). A blind randomization protocol was used. Animals were vaccinated with 0.5 ml as follows: for the standard vaccine, the vial was reconstituted with 7.5 ml of normal saline to make 15 doses of 0.5 ml each. The modified vaccine with an initial high *Mycoplasma* titre was reconstituted with 50 ml of PBS to make 100 doses of 0.5 ml each. The diluents in which the vaccines were reconstituted were placed on ice during reconstitution. This was carried out at the crush site where the animals were to be vaccinated.

Each of the 90 animals was subcutaneously inoculated with 0.5 ml of the standard vaccine, the modified vaccine or PBS at the caudal aspect of the tail or the tail-tip (Turner and Trethewie, 1961). After vaccination, the three cohorts were grazed and housed separately (between 200 and 500 m apart) for a period of 30 days after which they were managed as a single group. The thickness of the tail-tip (circumference of tail around the last coccyx) was measured daily in each animal for 30 days after inoculation to assess the occurrence of post-vaccinal reactions. Rectal temperatures were taken daily, between 08.00 and 10.00, with temperatures of 39.5 °C and above considered indicative of fever. Up to 3 months post vaccination, the animals were bled weekly for preparation of serum samples on which CFT and cELISA tests were carried out. After 3 months, half of the cattle from each cohort were randomly selected and treated as groups 1–6 (Table 1). Groups 1, 2 and 3 were challenged at 3 months, and those in groups 4, 5 and 6 were bled monthly thereafter until challenge at 16 months; weekly bleeding was resumed after challenge to monitor infection parameters.

### 2.4 Challenge and clinical examination

Challenge was carried out by endoscopic intubation using pathogenic *Mmm*SC cultures obtained from the Kenyan B237 isolate of *Mmm*SC. Briefly, each animal was intubated with a fibre-optic bronchoscope (VFS-2A, Swiss Precision, USA) through which 60 ml of *Mmm*SC culture containing 3 × 10^8^ colony forming units (cfu) of *Mmm*SC was introduced into the distal trachea, followed sequentially by 15 ml of 1.5% low temperature melting agar (36 °C) suspended in distilled water and 35 ml of PBS at 36 °C. The animals were observed daily for clinical signs for the period during which they were fed outdoors (09.00–15.30).

### 2.5 Necropsy and sample collection

Cattle showing fever for 10 days were euthanized on humane grounds, while those that did not were killed at 45 days post challenge. The animals were stunned and then exsanguinated before a full post-mortem examination was carried out.

### 2.6 Lesion scoring

The size of lung lesions (diameter in cm) was recorded and lung pathology scored according to the system proposed by Hudson and Turner (1963), in which the score is a combination of the size of the lesion and the isolation of the *Mmm*SC from tissues. We also calculated a score based solely on lung lesions as follows: grossly normal lungs, 0; fibrosis or evidence of disease remission, 1; presence of adhesions or consolidation, 2. Where sequestra were present, they were scored on the basis of size: less than 5 cm, 3; between 5 and 10 cm, 4; between 10 and 20 cm, 5; over 20 cm, 6. Protection rate (defined as the percentage reduction in lung pathology brought about by vaccination) was calculated from the lesion scores of control and vaccinated animals according to Hudson and Turner (1963), using the formula (NV − V) × 100/NV, where NV is the score of the non-vaccinated group and *V* is the score of the vaccinated group.

### 2.7 CFT and cELISA tests

The serum samples were tested using the cELISA test kit developed by CIRAD-EMVT, France, as described by the manufacturers. The optical density (OD) was measured at 450 nm with a plate reader (PR 2100, Sanofi, Pasteur diagnostic, US) and the percentage inhibition (PI) value for each serum sample calculated. A cut-off point of 50% inhibition was used to determine positive and negative samples, with a range between 45% and 55% being considered doubtful.

CFT was performed essentially according to the method of Campbell and Turner (1953). Briefly, the test employed commercially prepared complement and hemolysin (Biomereau®), and results were read after overnight incubation at 4 °C. Samples with a reading of 3 or 4 in a 1:10 dilution were re-titrated to obtain the end point titre. A reading of 2 or 1 at the 1:10 dilution was considered negative while readings of 4, 3 and 2 were considered positive.

### 2.8 Statistical analysis

On vaccine titration, analysis of variance (ANOVA) was used to determine if there were differences in titres between the two vaccines at different temperatures and periods. GenStat statistical software (9th edition) was used in the analysis. The difference between means in the variables (vaccine, temperature and time) was analyzed.

Comparative analysis of the changes in tail-tip circumferences and rectal temperatures between animals vaccinated with the standard vaccine, the modified vaccine, and the controls was performed using ANOVA. Pathology scores were evaluated to determine if there was a difference in protection afforded by the two vaccines. Differences between mean pathology scores in the three groups (vaccinates and controls) were also analyzed using ANOVA. To evaluate differences between the antibody response (as revealed by CFT and cELISA tests) induced by the two vaccines, a comparison of the proportions of the animals (vaccinates and controls) that responded was performed using Chi-square (*χ*^2^) test.

## 3 Results

### 3.1 Post vaccination responses

Daily mean tail-tip circumferences (in cm) of control (injection with PBS) and vaccinated cattle over 30 days post vaccination were collected. The observed changes arose either from circumscribed swellings or a bulb of variable diameter at the site of inoculation. The circumferences in individual animals differed from 3.5 cm for control animals to 5.1 cm for severely affected animals and varied between 3 and 21 days post-inoculation in both vaccinated groups. In 11 animals inoculated with the standard vaccine, oedema was markedly extended to the anterior aspect of the tail. Palpable swellings were rarely detectable before day 7 but in most cases became evident by day 14 post inoculation and regressed by day 21. In some animals, no obvious swelling appeared at the site of the vaccination. In the control group, swellings (probably attributable to trauma) were detected only for the first two days post vaccination.

In the analysis of variance, a comparison of the mean circumference for the vaccinated animals (4.66 ± 0.057 and 4.26 ± 0.059 for the standard and the modified vaccines, respectively) with that of the controls (3.35 ± 0.027) indicated that the vaccinates had significantly more swelling than the controls (*p* < 0.001). A comparison between the means for the vaccinated groups indicated that swellings in the standard group were greater than those in the modified group (*p* < 0.001). Rectal temperatures for all the animals remained within the normal range during the 30 days of post vaccination observation, suggesting that no serious infection was present (data not shown).

### 3.2 Post challenge clinical signs

Clinical responses following challenge at both 3 and 16 months post vaccination were typical of CBPP and included nasal discharge, cough, fever, labored breathing, disinclination to move and adoption of postures suggestive of oxygen deficiency. Animals with fever (temperature between 39.5 and 40.6 °C) also had anorexia. Fourteen of 44 animals challenged after 3 months showed fever, with the earliest raise in temperature observed on day seven post inoculation. Out of these, 4 (26.7%) were from the control group, 5/15 (33.3%) from the group that received the standard vaccine and 5/14 (35.7%) from the group that received modified vaccine. Following challenge at 16 months post vaccination, 21 of 44 cattle showed fever, with the earliest raise in temperature recorded on day four after challenge. Unvaccinated control animals all developed fever of at least 3 days duration between days 4 and 20 following challenge.

### 3.3 Necropsy and lesion scores

Table 2 summarizes the various descriptors of pathology and mortality as measured for the two challenge experiments at 3 and 16 months post vaccination. All parameters suggest a better outcome for animals from the two vaccinated groups.

The number of animals requiring euthanasia on humane grounds before the end of each challenge experiment was highest for the non-vaccinated control group: 9/15 animals for the 3 month challenge (compared to 3/15 for both standard and modified vaccines) and 13/15 animals for the 16 month challenge (compared to 2/15 for standard vaccine and 4/15 for the modified vaccine).

Isolation of *Mmm*SC was also about half as frequent in vaccinated cattle as in the non-vaccinated group for the 3 month challenge (3/15 and 5/14 compared to 9/15, respectively). This was not the case for the 16 month challenge, where numbers were equally high in the three groups (Table 2).

Post-mortem examination revealed gross pathological lesions characteristic of CBPP including consolidation of the lung parenchyma and pleuritis, and well-developed sequestra that were either unilateral or bilateral. In some cases the pleural cavity contained copious amounts (up to 5 l) of clear amber-colored fluid with fibrinous flecks. Fibrous adhesions of the parietal and visceral pleurae were also observed. Lung consolidation was accompanied by the hepatization and marbling appearance characteristic of CBPP.

**Fig. 1:**
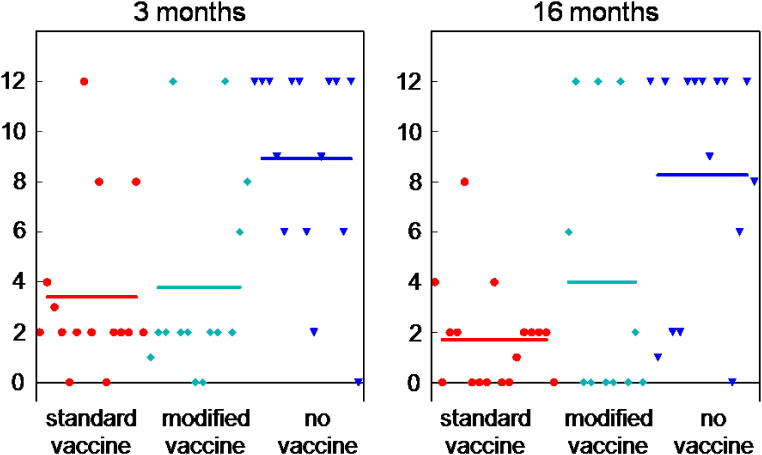
Lesion scores of individual cattle, calculated as a combination of lesion size and tissue isolation, challenged 3 or 16 months post vaccination (means represented by horizontal bars). Animals that received the standard vaccine (red dots), modified vaccine (cyan diamonds) or no vaccine (blue triangles). (For interpretation of the references to colour in this figure legend, the reader is referred to the web version of this article.).

The occurrence of lung lesions was twice as frequent in the non-vaccinated animals of both challenge groups. Average scores for lesion size (see Section 2.6) were also higher in non-vaccinated animals (Fig. 1 and Table 2).

Composite pathology scores were derived as described by Hudson and Turner (1963), on the basis of lesion occurrence, lesion size and isolation of *Mmm*SC from tissues. Mean scores for non-vaccinated groups were higher than those for vaccinated groups in both 3 and 16 month challenges. However, no significant difference was apparent between the two vaccinated groups in each case.

### 3.4 Serology post vaccination and before challenge

Serological responses to vaccination were measured by CFT and cELISA tests and are summarized in Table 2. Following vaccination, seroconversion by CFT was observed in 19/30 (63.3%) and 5/30 (16.7%) animals receiving the standard and the modified vaccines, respectively. Seroconversion by cELISA was observed in 5/30 (16.7%) and 4/29 (13.8%) of animals for the standard and modified groups, respectively. Seroconversion was first detected two weeks post vaccination, and cattle immunized with the standard vaccine seroconverted on average one week earlier than those receiving the modified formulation. As expected, seroconversion was not observed in the control group with either test up to 3 months post vaccination, the time of the first challenge. Comparison of cELISA antibody titres derived from animals vaccinated with the standard and modified vaccines with those of control animals confirmed that there was indeed a response in the vaccinates (*χ*^2^ = 5.62, *p* = 0.06 with 2 df)), but no difference was apparent between the animals vaccinated with the standard or modified vaccines (*χ*^2^ = 0.00, *p* = 1.000 with 1 df).

Challenge at 3 months post vaccination was carried out using half of the animals in each group. Those that remained to be challenged 16 months after vaccination were followed for seroconversion, observed by cELISA but not by CFT. Using a cut-off point of 55%, we found amongst the samples collected between 3 and 16 months post vaccination 63 positives out of a total of 403 samples in group 4 (standard vaccine), 41/404 in group 5 (modified vaccine) and only four false positives out of 387 samples in the non-vaccinated group 6, suggesting that the vaccinated groups had higher titres overall. However, no correlation could be found between the lesion scores after challenge and the cELISA titres before challenge, whether we used the mean cELISA titre over the whole 16 month period, the mean of the four samples from the month just before challenge, or the differences between the post- and pre-vaccination titres.

Analysis of the CFT antibody titres showed a statistically significant difference between vaccinated animals and control animals (*χ*^2^ = 33.07, *p* < 0.001 with 2 df). Within the vaccinated groups, the group that received the standard vaccine showed significantly higher (*χ*^2^ = 13.61, *p* < 0.001 with 1 df) titres than the group that received the modified vaccine.

### 3.5 Serology post-challenge

At 3 months post vaccination, seroconversion by CFT after challenge was observed in 21/44 cattle (Tables 2 and 3) (8/15 cattle vaccinated with standard vaccine, 5/14 with modified vaccine and 8/15 controls). Seroconversion by cELISA was observed in 24/44 cattle.

At 16 months post vaccination, seroconversion by CFT after challenge was observed in 20/44 cattle (Tables 2 and 3) comprising 5 out of 15 vaccinated with the standard vaccine, 8/14 with the modified vaccine and seven out of 15 controls. CFT titres ranged from 1/10 to 1/320. Almost all infected animals became sero-positive by cELISA after challenge.

### 3.6 Vaccine efficacy

Protection rate for the two vaccines, calculated using the average Hudson scores, are outlined in Table 2. After challenge at 3 months post vaccination, the protection rates for the standard and the modified vaccine were 77.1% and 51.8%, respectively. Similarly, following challenge at 16 months post vaccination, the rates of the conventional and the modified vaccines were 61.8% and 56.1%, respectively.

Comparative analysis of vaccinates and the controls showed that pathology was significantly reduced (*p* < 0.001) in vaccinated groups. However, the proportion of protected animals was not significantly different (*p* > 0.05) between the group vaccinated with the standard vaccine and that given the modified vaccine.

## 4 Discussion and conclusion

Our underlying hypothesis was that buffering the vaccine might improve the viability of *Mmm*SC and therefore allow larger numbers of live organisms to be delivered, resulting in improved immune responses. However, this was also expected to increase the possibility of post-vaccinal reactions at the site of inoculation. Temperature and tail-tip circumferences were therefore monitored. Reactions to both vaccines were similar and in line with observations from previous vaccination trials with the standard vaccine (Thiaucourt et al., 2000; Wesonga and Thiaucourt, 2000; Yaya et al., 1999). Rectal temperatures for all the animals remained within the normal range (no fever) during the 30 days post vaccination. However, analysis of variance of tail-tip circumferences between the animals vaccinated with the standard vaccine, the modified vaccine, and the PBS control animals indicated inflammation in vaccinates. A comparison indicated that the means for vaccinates were significantly higher than those of the controls (*p* < 0.001). Post-vaccinal reactions observed in this study were less severe than those reported in a previous study (Wesonga and Thiaucourt, 2000), where at least 1/40 cattle vaccinated with the T1/44 strain of *Mmm*SC developed a severe reaction necessitating destruction. This might reflect the fact that vaccination in that trial was carried out on the neck and not at the tail.

Seroconversion was observed during the 2 months after vaccination by both CFT and cELISA. However, throughout the 16 months of observation, the cELISA test returned significantly higher average titres in the vaccinated groups, particularly in the first two months. Serum titres measured by cELISA varied considerably between animals and between time of sampling, making identification of vaccinated animals only possible if numerous samples could be analyzed and compared with those from non-vaccinated animals. Since no correlation could be found between antibody levels and pathology (lesion score), it remains in question whether antibodies measured in cELISA are associated with protection. Although titrations for CFT antibodies were carried out, the trial protocol did not allow the animals to reach the end point of the disease progression.

A number of parameters confirmed that the vaccinated groups were protected, but little difference was apparent between the two vaccine preparations. In the non-vaccinated groups, more animals had fever, and three times as many were euthanized before the end of the experiment because of severe pathology, twice as many had lung lesions and more yielded *Mmm*SC from tissue (Table 2). Lesions of CBPP were similar to those observed in other studies, but were on average smaller in the two vaccinated groups than in the non-vaccinated group. However, no statistically significant differences were found between the groups vaccinated with the different vaccine preparations for any measured parameter. Although the study indicated that the standard vaccine offers a better protection (77%) than the modified vaccine (52%) after 3 months, the difference was not statistically significant (*p* > 0.05). The protection rates after 16 months were also very similar (62% and 56%). This demonstrates that the protection offered by the live vaccine preparations is still present more than one year after vaccination, which is longer than was generally accepted (Windsor et al., 1972; Gilbert et al., 1970; Masiga et al., 1978., Wesonga and Thiaucourt, 2000). However, this was a limited study carried out under optimal conditions. Variables that might affect vaccine efficacy include *Mmm*SC viability and quantity in the vaccine preparation, the condition of the animals (stress and other diseases) when vaccinated, and the conditions under which the vaccine must be administered.

The protection rates achieved in this experiment (between 52% and 77%) are comparable to those observed in previous studies and are in agreement with the statistical estimates of T_1_44 vaccine efficiency (Mariner et al., 2006a,b) of 50–80%. Trials carried out earlier (Thiaucourt et al., 2000; Yaya et al.,1999) established that a single vaccination conferred between 30% and 60% protection while a second boosting vaccination raises protection levels to 80–90% (Wesonga and Thiaucourt, 2000), suggesting that levels of protection consistent with control of the disease could be achieved with a second vaccination.

Despite observations *in vitro* (March, 2004), incorporation of a buffer in the culture medium did not improve efficacy of the vaccine in this clinical trial. In laboratory conditions, the time span between reconstitution and administration of the vaccine is relatively short, while in field conditions this is not necessarily the case. It is then also expected that the benefits of the buffered vaccine will be more visible in the field when less optimum conditions for preservation of viability are present. Large-scale field studies have been carried out in a CBPP-endemic area in Kenya to address this question (manuscript in preparation).

Our data suggest that improving the quality of viable organisms does not improve the efficacy of the vaccine under the conditions described. In previous studies, evaluation of the T1/44 or T1sr vaccines across a range of doses did not reveal a dose–response effect (Thiaucourt et al., 2000). Considering our data, this suggests that as long as a sufficient number of viable organisms are administered that have the potential to multiply, enough antigen will be present to induce an immune response.

## Figures and Tables

**Table 1:** Ultimate groupings of experimental cattle at the time of challenge, and number of animals that provided data.

Group	Number of animals	Immunizing inoculum	Challenge
1	15	Standard	3 months
2	14	Modified	3 months
3	15	PBS	3 months
4	15	Standard	16 months
5	14	Modified	16 months
6	15	PBS	16 months

**Table 2:** Infection and pathology parameters after challenge of vaccinated and non-vaccinated groups, 3 or 16 months after vaccination.

Challenge post vaccination	3 months			16 months		
Vaccination group	Standard	Modified	PBS	Standard	Modified	PBS
Number of cattle	15	14	15	15	14	15
Average number of fever days post challenge	1.5	3.1	4.9	1.3	3	8.5
% Euthanised	20%	29%	60%	2%	4%	13%
% With *Mycoplasma* isolated	20%	36%	67%	73%	64%	73%
% With lesions	60%	43%	93%	40%	43%	87%
Average lesion score^a^	2.7 ± 2.8	2.7 ± 2.6	4.7 ± 1.6	1.9 ± 2.2	2.6 ± 2.4	5.3 ± 1.8
Average Hudson score^b^	1.9 ± 2.2	4.0 ± 5.5	8.3 ± 4.8	3.4 ± 3.3	3.8 ± 4.1	8.9 ± 4.0
% Seroconverted CFT	53%	36%	53%	33%	53%	27%
% Seroconverted cELISA	64%	46%	31%	100%	100%	87%
Vaccine efficacy	77%	52%	–	62%	56%	–

a Lesion score calculated from the size of lung lesions.

b Lesion score calculated as a combination of lung lesion and tissue isolation of *Mycoplasma* as in Hudson and Turner (1963).

**Table 3:** Serology of animals after vaccination and after challenge.

	CFT^a^			c-ELISA^b^		
	No vaccine	Modified	Standard	No vaccine	Modified	Standard
Post vaccination						
% +ve animals seroconverted	0%	17%	63%	0%	14%	17%
% +ve samples^c^ between 3 and 16 months	0.53%	0%	0.74%	1.03%	9.4%	15%
3 month challenge						
% +ve animals after challenge	53%	36%	53%	31%	46%	64%
16 month challenge						
% +ve animals after challenge	50%	53%	33%	100%	100%	87%

a Complement fixation test.

b Competitive ELISA (Le Goff and Thiaucourt, 1998).

c Each animal was tested on 29 occasions between 3 and 16 months after vaccination (see Section 2 for details).
